# A Pilot Proteomic Analysis of Tear Fluid in Domestic Cats with and Without Conjunctivitis Using MALDI–TOF/TOF Mass Spectrometry

**DOI:** 10.3390/ani16060912

**Published:** 2026-03-13

**Authors:** Takuya Yogo, Shotaro Iino, Kinya Katayama

**Affiliations:** 1 Laboratory of Veterinary Surgery, Nippon Veterinary and Life Science University, Musashino 180-8602, Japan; 2 Laboratory of Biomolecular Chemistry, Nippon Veterinary and Life Science University, Musashino 180-8602, Japan

**Keywords:** feline tear secretion, conjunctivitis, proteomics, MALDI-TOF/TOF MS

## Abstract

Feline conjunctivitis is a common ocular disorder; however, the molecular composition of feline tear fluid and its alterations during ocular surface inflammation remain poorly characterized. This pilot study aimed to explore the tear proteome of cats with conjunctivitis using matrix-assisted laser desorption/ionization time-of-flight tandem mass spectrometry (MALDI–TOF/TOF MS) and to compare findings with those from clinically healthy cats. Tear samples were collected using Schirmer tear test strips from healthy cats and cats diagnosed with conjunctivitis. Total protein concentration was measured by ultraviolet absorbance spectrophotometry, and tear proteins were separated by SDS–polyacrylamide gel electrophoresis, followed by in-gel trypsin digestion and MALDI–TOF/TOF MS analysis. Nine distinct tear proteins were identified, including antimicrobial and immune-related components such as lactoperoxidase, lactotransferrin, albumin, and immunoglobulin A constant region. Lactoperoxidase and SBP1 were identified in feline tear fluid for the first time. No proteins uniquely associated with conjunctivitis were detected. The mean total tear protein concentration was numerically higher in cats with conjunctivitis (13.06 ± 0.75 mg/mL) than in healthy cats (9.69 ± 0.67 mg/mL); however, this difference did not reach statistical significance (*p* = 0.095) and should be interpreted cautiously given the limited sample size. This pilot study provides preliminary insights into tear protein profiles in cats with conjunctivitis and highlights the need for larger quantitative investigations. These findings provide a preliminary framework for future studies aimed at further characterizing molecular alterations associated with feline ocular surface disorders.

## Simple Summary

Conjunctivitis is a common eye condition in cats; however, the biological changes that occur in tear fluid during inflammation are poorly understood. Tears contain many proteins that protect the ocular surface, and changes in these proteins may reflect disease processes. In this pilot study, we compared tear samples from healthy cats and cats with conjunctivitis. Tears were collected using Schirmer tear test strips, a method routinely used in veterinary clinics. Protein composition was analyzed using gel electrophoresis and a mass spectrometry technique that allows for identification of proteins from very small sample volumes. This method involves measuring the masses of protein fragments and matching them to known proteins in databases. Several proteins associated with immune defense and ocular surface protection were identified in feline tears. Some of these proteins are well characterized in other species, whereas others were detected in feline tears for the first time. Although no proteins were detected exclusively in cats with conjunctivitis, the mean total tear protein concentration was higher in affected cats; however, this difference was not statistically significant. These findings expand current knowledge of tear proteins in cats and highlight the need for larger quantitative investigations. A better understanding of tear protein profiles may support the development of noninvasive diagnostic tools and improved treatment strategies for feline eye diseases.

## 1. Introduction

The tear film plays a crucial role in maintaining ocular surface health by providing lubrication, antimicrobial defense, and immunological protection. In mammals, tear fluid contains a complex mixture of proteins, lipids, electrolytes, and metabolites that collectively contribute to ocular surface stability and the regulation of local immune responses. Alterations in tear composition have been associated with several ocular surface disorders, including conjunctivitis, keratitis, and dry eye disease. Consequently tear fluid has attracted increasing interest as a noninvasive source of molecular indicators in human and veterinary ophthalmology [1,2].

Proteomic analyses of tear fluid have been extensively performed in humans and, to a lesser extent, in dogs. Previous studies have identified numerous tear proteins involved in antimicrobial activity, oxidative stress regulation, and immune modulation, including lactoferrin, lysozyme, immunoglobulins, and complement-related proteins [3]. In canine ophthalmology, tear proteomics has provided insights into breed-related differences, inflammatory ocular diseases, and tear film dysfunction, highlighting the diagnostic potential of tear-derived protein profiles [4]. In contrast, the molecular composition of feline tear fluid remains poorly characterized, and systematic proteomic studies in cats are limited.

Conjunctivitis is among the most common ocular surface disorders in cats and may arise from infectious, allergic, or idiopathic causes. In clinical practice, feline conjunctivitis is most commonly associated with infectious etiologies such as feline herpesvirus type 1 (FHV-1) [5], *Chlamydia felis*, and *Mycoplasma* spp., although secondary bacterial infections may also occur. Non-infectious causes include allergic conjunctivitis, immune-mediated inflammation, environmental irritation, trauma, and nasolacrimal duct abnormalities. Conjunctivitis may also accompany systemic diseases or represent an extension of corneal or adnexal pathology. Additionally, conjunctivitis may occur in association with eosinophilic keratoconjunctivitis, chronic rhinosinusitis or nasolacrimal disease, and as part of the feline respiratory disease complex. Clinically, feline conjunctivitis is characterized by conjunctival hyperemia, chemosis, and ocular discharge, reflecting inflammation of the conjunctival tissues. Although the clinical features and etiologies of feline conjunctivitis are well described in veterinary ophthalmology references [6], the molecular changes that occur within the tear film during conjunctival inflammation remain largely unknown. Characterizing these changes may provide a better understanding of disease mechanisms and inform the development of objective diagnostic or prognostic tools.

Matrix-assisted laser desorption/ionization time-of-flight tandem mass spectrometry (MALDI–TOF/TOF MS) has emerged as a practical platform for proteomic analysis of low-volume biological samples. This technique enables protein identification from microgram quantities and has been successfully applied to tear fluid analysis in humans and animals. However, its application in feline tear proteomics, particularly in the context of ocular surface disease, has not been systematically explored [7,8].

Therefore, this study aimed to explore tear protein profiles in cats with clinically diagnosed conjunctivitis using MALDI–TOF/TOF MS and to compare them with those from clinically healthy cats. We hypothesized that clinically diagnosed conjunctivitis is associated with detectable alterations in tear protein composition and/or total protein concentration compared with healthy cats. This investigation focused on clinically defined conjunctivitis rather than pathogen-specific subtypes, reflecting cases commonly encountered in routine veterinary practice. Total tear protein concentration was also assessed to evaluate potential quantitative changes associated with conjunctival inflammation. By offering an initial characterization of the feline tear proteome, this study serves a preliminary basis for future quantitative and disease-specific investigations of ocular surface disorders in cats.

## 2. Materials and Methods

### 2.1. Animals

#### 2.1.1. Inclusion and Exclusion Criteria

Healthy Group:

Clinically healthy cats were enrolled based on the following criteria:

(i) Negative test results for feline immunodeficiency virus (FIV) and feline leukemia virus (FeLV) using a commercial antigen/antibody test kit (SNAP Combo Plus; IDEXX Laboratories, Westbrook, ME, USA);
(ii) Negative polymerase chain reaction results for FHV-1 and *Chlamydia felis*;
(iii) Absence of clinical signs of ocular surface disease including conjunctival hyperemia, chemosis, ocular discharge, or corneal opacity;
(iv) Normal tear production (>15 mm/min, Schirmer tear test I) and no corneal epithelial defects confirmed by fluorescein staining.

Only eyes without conjunctival edema or corneal ulceration were included.

Conjunctivitis Group:

Cats with conjunctivitis were defined as those exhibiting marked conjunctival hyperemia and moderate to severe chemosis without corneal ulceration, as confirmed by fluorescein staining (Ayumi Pharmaceutical Corporation, Tokyo, Japan).

All affected cats tested negative for FIV, FeLV, FHV-1, and *Chlamydia felis* using the same diagnostic assays described above. Cats were excluded if they had corneal ulceration, keratitis, uveitis, systemic illness, or a history of topical ophthalmic medication use within 2 weeks prior to sampling.

Study Population

A total of ten domestic cats were included: five clinically healthy cats (three neutered males and two intact males; age range: 136–153 months; mean age: 147 months) and five cats diagnosed with conjunctivitis (four intact males and one intact female; age range: 117–158 months; mean age: 135 months) (Table 1).

A total of ten domestic cats (five clinically healthy cats and five cats diagnosed with conjunctivitis) were recruited for this study.

The number of eyes included differed between the total protein concentration analysis and the proteomic analysis because tear volume varied substantially among eyes.

For total protein concentration analysis, tear samples from 8 eyes of healthy cats and 10 eyes of cats with conjunctivitis were analyzed. Two eyes from healthy cats were excluded because excessive patient movement prevented adequate tear collection; sampling was discontinued in these cases to minimize animal stress.

For proteomic analysis (MALDI–TOF/TOF-MS), tear samples from nine eyes of five healthy cats (N1–5) were analyzed. Although six healthy cats were initially enrolled, samples from some eyes were excluded because of insufficient tear volume resulting from resistance to handling.

All cats were housed at the experimental animal facility of Nippon Veterinary and Life Science University. All examinations and sample collections were performed by the same veterinarian (T.Y.) in accordance with the approved institutional animal care and use protocol.

Before tear sampling, all cats underwent slit-lamp biomicroscopy (SL-15; Kowa Optimed Inc., Tokyo, Japan) and fluorescein staining to confirm the absence of corneal epithelial defects or ulceration. Cats exhibiting conjunctival hyperemia and moderate to severe conjunctival edema were assigned to the conjunctivitis group.

All experimental procedures were approved by the Institutional Animal Care and Use Committee of Nippon Veterinary and Life Science University (IACUC approval No. 2022S45) and conducted in accordance with the university’s ethical guidelines for animal experimentation.

Tear Collection

Tear fluid was collected without topical anesthesia using sterile Schirmer tear test strips (Schirmer Tear Test; MSD Animal Health, Tokyo, Japan). The strip was gently inserted into the lower conjunctival sac until the tear fluid reached the 30-mm mark. After collection, the strips were immediately stored at −80 °C until analysis.

#### 2.1.2. Recovery of Tear Fluid from Schirmer Tear Test Strips

After thawing on ice, each Schirmer tear test strip was placed into a nested microtube system consisting of an outer 1.5-mL tube (Eppendorf, Hamburg, Germany) and an inner 0.5-mL tube (Applied Biosystems, Thermo Fisher Scientific, Waltham, MA, USA) with a small hole created at the bottom using an 18-gauge needle.

Samples were centrifuged three times at 4 °C and 3000× *g* for 5 min (KUBOTA 3740; Kubota Co., Tokyo, Japan). During the second and third centrifugation steps, phosphate-buffered saline (PBS) equivalent to half of the estimated tear volume was added.

Based on the approximation that a 2-mm segment of the strip absorbs approximately 1 µL of tear fluid, a fully wetted 30-mm strip was estimated to contain ~15 µL of tear fluid. Accordingly, 7.5 µL of PBS was added during each elution step.

#### 2.1.3. Total Protein Concentration Measurement

The total protein concentration in tear samples was measured by ultraviolet (UV) absorbance spectrophotometry using a microvolume spectrophotometer (BioSpec-nano; Shimadzu Biotech, Kyoto, Japan). Absorbance was measured at 280 nm. Tear eluates recovered from Schirmer tear test strips were analyzed directly, and protein concentrations were calculated according to the manufacturer’s instructions.

#### 2.1.4. SDS–Polyacrylamide Gel Electrophoresis (SDS–PAGE)

For SDS–PAGE, protein concentrations determined by UV absorbance spectrophotometry were used to adjust sample loading to 10–50 µg per lane. Samples were diluted with ultrapure water and mixed with Laemmli sample buffer (Bio-Rad, Hercules, CA, USA) containing 5% β-mercaptoethanol at a ratio of 19:1. The mixture (10 µL sample + 10 µL buffer) was vortexed and heat-denatured at 100 °C for 5–10 min.

Protein separation was performed using precast 12.5% polyacrylamide gels (SuperSep^TM^ Ace, 13-well; Fujifilm Wako, Osaka, Japan) at 100 V, 20 mA, and 8 W for 1 h. Prestained molecular weight markers (Prestained SDS–PAGE Standards, Broad Range; Bio-Rad) were used as references. After electrophoresis, gels were rinsed with ultrapure water, stained with Quick-CBB (Fujifilm Wako Pure Chemical Industries, Osaka, Japan) for 30 min with gentle agitation, and destained overnight with repeated washes in ultrapure water.

#### 2.1.5. In-Gel Trypsin Digestion

Protein bands of interest were excised using a sterile scalpel blade (FEATHER Surgical Blade No. 11; Feather Safety Razor Co., Osaka, Japan) and processed using an In-Gel Tryptic Digestion Kit (Thermo Scientific, Waltham, MA, USA) according to the manufacturer’s instructions.

Briefly, gel pieces (~1 mm^3^) were destained in 200 µL of destaining solution (80 mg of ammonium bicarbonate in 20 mL of acetonitrile and 20 mL of Milli-Q water) at 37 °C for 30 min with shaking. Reduction was performed with 30 µL of reducing buffer (TCEP in digestion buffer) at 60 °C for 10 min, followed by alkylation with 30 µL of alkylation buffer (IAA in digestion buffer) at 20–22 °C for 60 min. After dehydration with acetonitrile, gel pieces were rehydrated with 10 µL of activated trypsin solution for 15 min and incubated in 25 µL of digestion buffer at 37 °C for 4 h or overnight at 30 °C. The resulting peptide solutions were collected for mass spectrometric analysis.

#### 2.1.6. MALDI–TOF/TOF MS and Database Search

Peptide mass fingerprinting and tandem mass spectrometry (LIFT mode) were performed using a MALDI–TOF/TOF MS (autoflex speed TOF/TOF; Bruker Daltonics, Billerica, MA, USA). The resulting tryptic peptides were desalted and concentrated using ZipTip C18 (Millipore, Burlington, MA, USA), and eluted onto a MALDI target plate with 2 µL of matrix solution (saturated α-cyano-4-hydroxycinnamic acid in 50% acetonitrile, 0.1% trifluoroacetic acid). After air-drying, spectra were acquired in positive reflector mode. External mass calibration was performed using a Peptide Calibration Standard Kit (Bruker Daltonics, Billerica, MA, USA), and trypsin autolysis peaks were excluded prior to database searching.

Protein identification was conducted using the MS-Fit search engine implemented in Protein Prospector (version 6.4.9; University of California, San Francisco, CA, USA; https://prospector.ucsf.edu). Database searches were performed against UniProtKB (release 2 September 2020; https://www.uniprot.org) or Swiss-Prot (release April 2023), with taxonomy restricted to *Felis catus*. Trypsin was specified as the proteolytic enzyme, and up to one missed cleavage was allowed. Mass tolerance was set to 100 ppm, and the instrument type was specified as MALDI–TOF/TOF. Carbamidomethylation of cysteine was selected as a fixed modification, and oxidation of methionine was specified as a variable modification. All other parameters were set to default values.

### 2.2. Statistical Analysis

Total protein concentrations in tear samples were compared between healthy cats and cats with conjunctivitis using the Mann–Whitney U test. A nonparametric test was selected owing to the small sample size and non-normal data distribution. Statistical significance was set at *p* < 0.05. Analyses were conducted using GraphPad Prism (version 9.0; GraphPad Software, San Diego, CA, USA).

### 2.3. Ethical Approval Statement

All experimental procedures involving animals were reviewed and approved by the Institutional Animal Care and Use Committee of Nippon Veterinary and Life Science University (IACUC approval No. 2022S45) and conducted in accordance with institutional guidelines for the ethical use of animals in research.

## 3. Results

### 3.1. Tear Collection

Tear fluid was successfully collected from 18 eyes (8 from healthy cats and 10 from cats with conjunctivitis) using Schirmer tear test strips. Fluorescein staining confirmed the absence of corneal epithelial defects in all included eyes.

### 3.2. Protein Concentration in Tear Samples

Total protein concentrations measured by UV absorbance spectrophotometry were 13.06 ± 0.75 mg/mL (*n* = 10) and 9.69 ± 0.67 mg/mL (*n* = 8) in cats with conjunctivitis and healthy cats, respectively (mean ± SD; Supplementary Table S1). This difference was not statistically significant (Mann–Whitney U test, *p* = 0.095). Given the exploratory design of this pilot study, these findings should be interpreted cautiously.

### 3.3. SDS–PAGE Analysis

SDS–PAGE analysis revealed no protein bands uniquely present in cats with conjunctivitis. Overall, tear protein profiles appeared largely comparable between healthy cats and cats with conjunctivitis, indicating no disease-specific qualitative changes detectable by gel-based analysis.

Detailed SDS–PAGE results, including reproducibility assessments and representative gel images, are provided in the Supplementary Materials (Figures S1–S4).

### 3.4. Protein Identification by MALDI–TOF/TOF MS

Among 14 excised SDS–PAGE bands, 9 distinct proteins were identified by MALDI–TOF/TOF MS. Initial protein identification was based on peptide mass fingerprinting, and selected peptides were further validated by tandem mass spectrometry (LIFT mode) when signal intensity permitted. The identified proteins included lactoperoxidase, lactotransferrin, serum albumin, the IgA constant region, selenium-binding protein 1 (SBP1), and Fel d 7 allergen (Supplementary Table S2). Lactoperoxidase and SBP1 have not been previously reported in the feline tear proteome. A summary of protein identifications, including UniProt accession numbers, peptide matching rates, and functional annotations, is presented in Supplementary Table S2. Representative peptide mass spectra and MS/MS confirmation data are shown in Supplementary Figures S5–S13.

### 3.5. Presence or Absence of the Identified Tear Proteins

The presence or absence of the identified tear proteins in healthy cats and cats with conjunctivitis is summarized in Table 2. Detailed protein identification data including peptide matching rates and representative mass spectra are provided in Supplementary Table S2 and Supplementary Figures S5–S13.

As shown in Table 2, lactoperoxidase, albumin, lactotransferrin, and the IgA constant region were detected in both healthy cats and cats with conjunctivitis, consistent with major baseline components of mammalian tear fluid. In contrast, SBP1, Fel d 7 allergen, and a scavenger receptor cysteine-rich (SRCR) domain-containing protein were detected in the conjunctivitis group but not in the healthy group. Because this pilot study employed a qualitative, abundance-biased platform and included a limited cohort, non-detection should not be interpreted as true absence of these proteins in healthy cats, nor as evidence of disease specificity.

## 4. Discussion

This pilot study is one of the first proteomic investigations of feline tear fluid using MALDI–TOF/TOF MS. Nine distinct tear proteins were identified including components previously reported in human and canine tears as well as proteins not previously described in feline tear fluid, such as lactoperoxidase and SBP1. Collectively, these findings provide preliminary insights into the molecular composition of feline tears and describe proteins detected in the context of ocular surface inflammation.

### 4.1. Tear Collection Methodology

Schirmer tear test strips enabled reliable, noninvasive tear collection from conscious cats. This approach provides higher protein recovery and more consistent sampling volumes than microcapillary techniques [9].

In this study, preliminary evaluation indicated that the blue dye present on the test strips did not substantially interfere with UV absorbance measurements, supporting the suitability of this method for quantitative and proteomic analyses. These observations suggest that Schirmer tear test strip-based sampling is practical for future clinical investigations in feline ophthalmology.

### 4.2. Tear Protein Concentration and Inflammatory Changes

Although total protein concentration was numerically higher in conjunctivitis tear samples than in healthy controls, the difference was not statistically significant. Given the limited sample size and exploratory design, this finding should be interpreted cautiously. In human ocular surface disorders, increased tear protein levels have been associated with increased vascular permeability and immune activation [3]. In this study, the absence of qualitative differences by SDS-PAGE suggests that conjunctival inflammation may primarily affect protein abundance rather than induce novel protein expression. In feline eyes, conjunctival inflammation may influence tear film composition through vascular leakage and local immune activation, as suggested by experimental and clinical studies of ocular surface inflammation [1,2]. However, because the underlying etiology and disease stage were not defined in the present cohort, these interpretations remain speculative. No conjunctivitis-specific protein bands were detected, indicating that the observed changes were predominantly quantitative rather than qualitative.

Given the exploratory design of this study and the limited sample size, more sensitive quantitative proteomic approaches will be required to clarify subtle changes in tear protein abundance.

### 4.3. Proteins Identified by MALDI–TOF/TOF MS

This study is the first to identify lactoperoxidase in feline tear fluid. As a heme peroxidase with established antimicrobial activity in human mucosal secretions [10,11], lactoperoxidase may contribute to innate immune defense at the feline ocular surface. However, its specific functional role in feline tears remains to be determined. Its detection expands the current descriptive profile of the feline tear proteome.

SBP1 was also identified for the first time in feline tears. SBP1 is implicated in redox regulation and cellular differentiation [12]. While its specific function in tear fluid remains unclear, its detection suggests that oxidative stress-related pathways may be represented in the feline tear proteome and warrants further investigation.

Fel d 7 allergen, a lipocalin protein recognized as a major feline allergen, was also identified, which further supports species-specific tear protein expression [13,14]. Lipocalins participate in immune modulation at mucosal surfaces, and their detection in feline tears highlights the immunological complexity of the ocular surface environment. In addition, an SRCR domain-containing protein was identified. Related SRCR proteins have been reported in canine tear fluid [4], and are associated with innate and mucosal immune processes in other species [15,16]. While these parallels may suggest conservation of immune-related components across species, the functional relevance of these proteins in feline ocular surface immunity requires further investigation.

### 4.4. Future Directions

To build upon these preliminary observations, future studies may benefit from the following approaches:

1. Applying quantitative proteomic techniques, such as LC–MS/MS or SWATH-MS, to detect subtle changes in tear protein abundance.
2. Including larger, etiologically defined cohorts that incorporate microbiological and cytological characterization of conjunctivitis.
3. Correlating tear proteomic profiles with clinical disease severity and therapeutic response.
4. Integrating proteomic data with metabolomic and cytokine profiling to achieve comprehensive molecular characterization of feline ocular surface disorders.

These approaches may facilitate the evaluation of candidate tear protein signatures and further clarify molecular mechanisms underlying feline ocular surface disease.

### 4.5. Methodological Considerations and Limitations

This study was designed as an exploratory pilot study and was not powered to detect subtle quantitative differences between groups. The small sample size (*n* = 5 per group) limited statistical power and precluded definitive conclusions regarding disease-associated proteomic alterations. Although the mean total tear protein concentration was numerically higher in cats with conjunctivitis (*p* = 0.095), this finding should be interpreted cautiously given the limited cohort size.

MALDI–TOF/TOF MS was selected as an appropriate qualitative platform for this exploratory investigation because it enables reliable protein identification from limited tear volumes using a relatively simple workflow. However, this approach provides lower proteome depth than LC–MS/MS-based quantitative platforms and is inherently biased toward the detection of relatively abundant proteins. Consequently, low-abundance proteins or extensively modified proteoforms might not have been detected. LC–MS/MS platforms offer superior sensitivity and quantitative accuracy for comprehensive tear proteome profiling [7,17,18]. Recent UHPLC–MS/MS workflows and data-independent acquisition strategies have further enhanced analytical depth and reproducibility [17,18]. Although direct comparative evaluations between LC–MS/MS and MALDI–TOF/TOF platforms in tear proteomics remain limited, existing literature indicates that these platforms yield distinct analytical profiles.

Immunoassay-based approaches remain useful for targeted analyses but are constrained by antibody specificity and limited dynamic range [19,20].

Microbiological and molecular characterization of conjunctivitis etiology was not performed, and standardized clinical severity scoring was not applied. Therefore, the present findings represent clinically diagnosed conjunctivitis rather than etiologically defined subtypes. Because disease stage and underlying etiology may influence tear protein composition, future investigations incorporating pathogen identification and severity stratification will be necessary to determine whether distinct proteomic patterns exist among clinical subgroups.

## 5. Conclusions and Perspectives

This exploratory proteomic analysis indicates that feline tear secretion contains numerous proteins associated with immune defense and redox regulation. The identification of lactoperoxidase and SBP1 as previously unreported components of feline tear fluid expands the current descriptive profile of the feline tear proteome and highlights candidate proteins for future quantitative and functional investigation.

This pilot study represents one of the first proteomic characterizations of tear fluid in cats with conjunctivitis using MALDI–TOF/TOF MS. Nine distinct proteins, including previously unreported components in feline tear fluid such as lactoperoxidase and SBP1, were identified. While no disease-specific proteins were detected by gel-based analysis, these findings provide a preliminary reference point for future quantitative proteomic studies aimed at evaluating protein alterations in feline ocular surface disorders. The observed difference in total tear protein concentration between healthy cats and those with conjunctivitis did not reach statistical significance and should be interpreted cautiously given the limited sample size.

Future research should employ high-resolution quantitative proteomic approaches, such as label-free LC–MS/MS or data-independent acquisition strategies, to clarify subtle changes in individual tear protein abundance. Integrating proteomic data with clinical parameters, cytokine profiling, microbiological diagnostics, and metabolomic analyses may further elucidate molecular pathways involved in feline ocular surface disorders.

In conclusion, this pilot study provides an initial qualitative characterization of the feline tear proteome in cats with conjunctivitis. Importantly, the combination of Schirmer tear test strip sampling and MALDI–TOF/TOF MS demonstrates the feasibility of proteomic profiling using minimally invasive tear collection methods applicable in routine veterinary practice. Although constrained by small sample size and the qualitative, abundance-biased nature of the analytical platform, this study provides a preliminary framework for future quantitative, etiology-specific, and longitudinal investigations. From a clinical standpoint, this workflow offers several practical advantages: (i) minimally invasive tear collection using routinely available Schirmer tear test strips; (ii) qualitative protein identification from small tear volumes using a simplified analytical workflow; and (iii) an exploratory screening platform to guide subsequent high-resolution quantitative LC–MS/MS investigations aimed at advancing noninvasive diagnostic strategies in veterinary ophthalmology.

## Figures and Tables

**Table 1:** Healthy cats and cats with conjunctivitis included in the study.

Healthy Cats		
Cat ID	Sex	Age (Months)
N1	Neutered male	144
N2	Neutered male	136
N3	Intact male	153
N4	Intact male	152
N5	Neutered male	150
	Mean age	147
**Cats with Conjunctivitis**		
**Cat ID**	**Sex**	**Age (Months)**
CJ1	Intact male	158
CJ2	Intact male	117
CJ3	Intact male	117
CJ4	Intact male	124
CJ5	Intact female	158
	Mean age	135

Note: Sex and age (in months) are shown for each cat. Cats were assigned to the healthy or conjunctivitis group based on findings from clinical ophthalmic examinations. Reproductive status (neutered and intact) is indicated.

**Table 2:** Presence or absence of tear proteins identified in healthy cats and cats with conjunctivitis.

Protein Name	Healthy Cats	Conjunctivitis Cats
Lactoperoxidase	+	+
Albumin	+	+
Lactotransferrin	+	+
IgA constant region	+	+
Selenium-binding protein 1	−	+
Fel d 7 allergen	−	+
SRCR domain-containing protein	−	+

Note: “+” indicates protein detection by MALDI–TOF/TOF MS, whereas “−” indicates non-detection. Given the qualitative and abundance-based nature of gel-based MALDI–TOF/TOF-MS, “−“ does not exclude the presence of the protein at lower abundance or outside the detectable range. Protein identification was based on peptide mass fingerprinting and tandem mass spectrometry following SDS–PAGE separation. Detailed identification data, including UniProt accession numbers and peptide matching rates, are provided in Supplementary Table S2.

## Data Availability

The original contributions presented in this study are included in the article/Supplementary Material. Further inquiries can be directed to the corresponding author.

## Supplementary Materials

The following supporting information can be downloaded at: https://www.mdpi.com/article/10.3390/ani16060912/s1; The Supplementary Materials include detailed tear protein concentration data (Table S1), extended SDS–PAGE results (Figures S1–S4), representative MALDI–TOF/TOF MS results for protein identification (Figures S5–S13), and a comprehensive list of identified tear proteins with functional annotations (Table S2). No measurable difference in absorbance at 280 nm was observed between PBS alone and PBS eluted from unused Schirmer tear test strips.
