# Supplementary material for: A Pilot Proteomic Analysis of Tear Fluid in Domestic Cats with and Without Conjunctivitis Using MALDI–TOF/TOF Mass Spectrometry
Source: Animals (Basel). 2026 Mar 13;16(6):912. doi: 10.3390/ani16060912 (PMC13023246; doi:10.3390/ani16060912)
Supplement: Supplementary file 1 [file animals-16-00912-s001.zip › animals-4133073-supplementary.pdf]

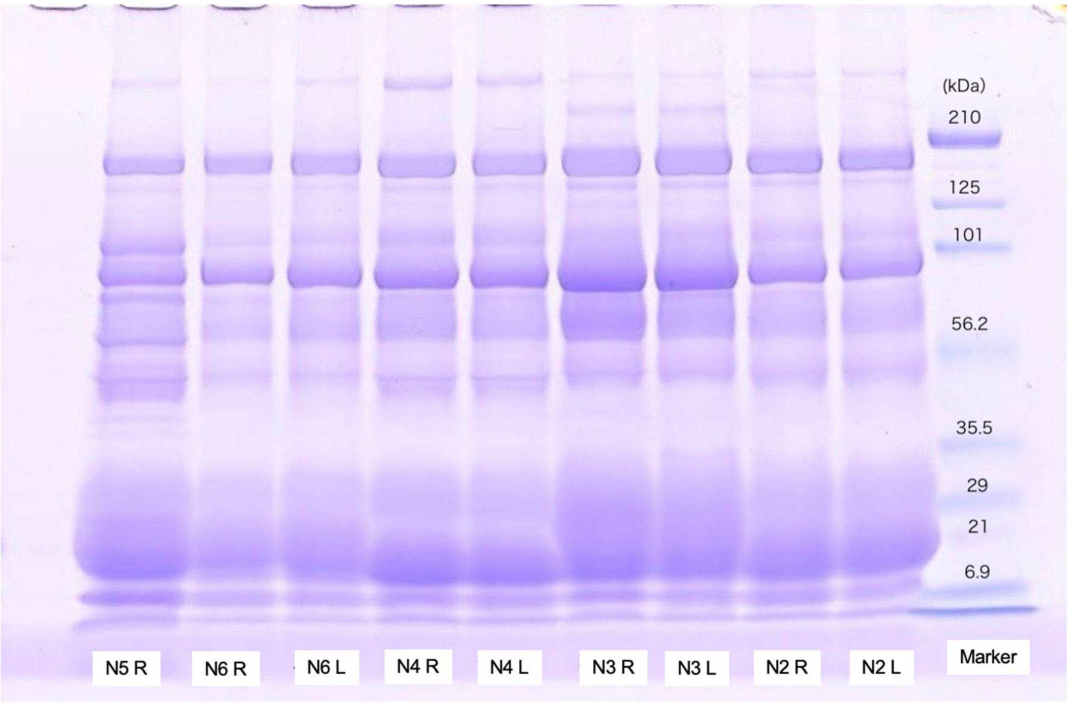

Figure S1. SDS–PAGE analysis of tear proteins from healthy cats

Figure S1. Representative sodium dodecyl sulfate–polyacrylamide gel electrophoresis (SDS–PAGE) profiles of tear protein samples obtained from healthy cats. Each lane corresponds to an individual tear sample, with the sample identifiers indicated below the lanes. The abbreviations “L” and “R” denote left and right eyes, respectively. The rightmost lane contains a prestained molecular weight marker (BIO-RAD, Prestained SDS–PAGE Standards, Broad Range), and the apparent molecular weights (kDa) of the marker bands are indicated. Protein bands were visualized by Coomassie Brilliant Blue staining. The gel image is shown as a representative result of three independent SDS–PAGE runs.

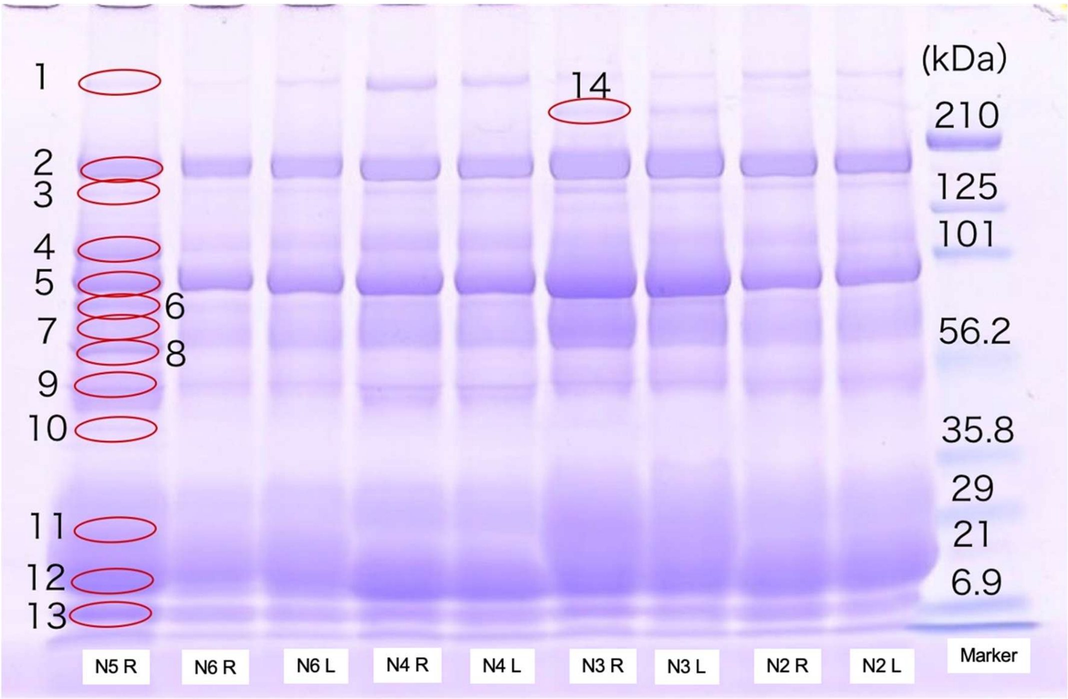

Figure S2. Excision of protein bands from SDS–PAGE gels of healthy cat tear samples for mass spectrometric analysis

Figure S2. Protein bands (Bands 1–14) excised from SDS–PAGE gels of tear samples obtained from healthy cats for subsequent mass spectrometric identification. The bands selected for excision are indicated by red circles. Bands corresponding to those identified in the sample from N5 R were excised from all other lanes in which the same bands were present and were subjected to mass spectrometry under identical conditions. In addition, a high–molecular-weight band observed at approximately 210 kDa in the sample from N3 was excised from both the right (R) and left (L) eye lanes and analyzed separately by mass spectrometry. Band numbering corresponds to the positions indicated in the gel image.

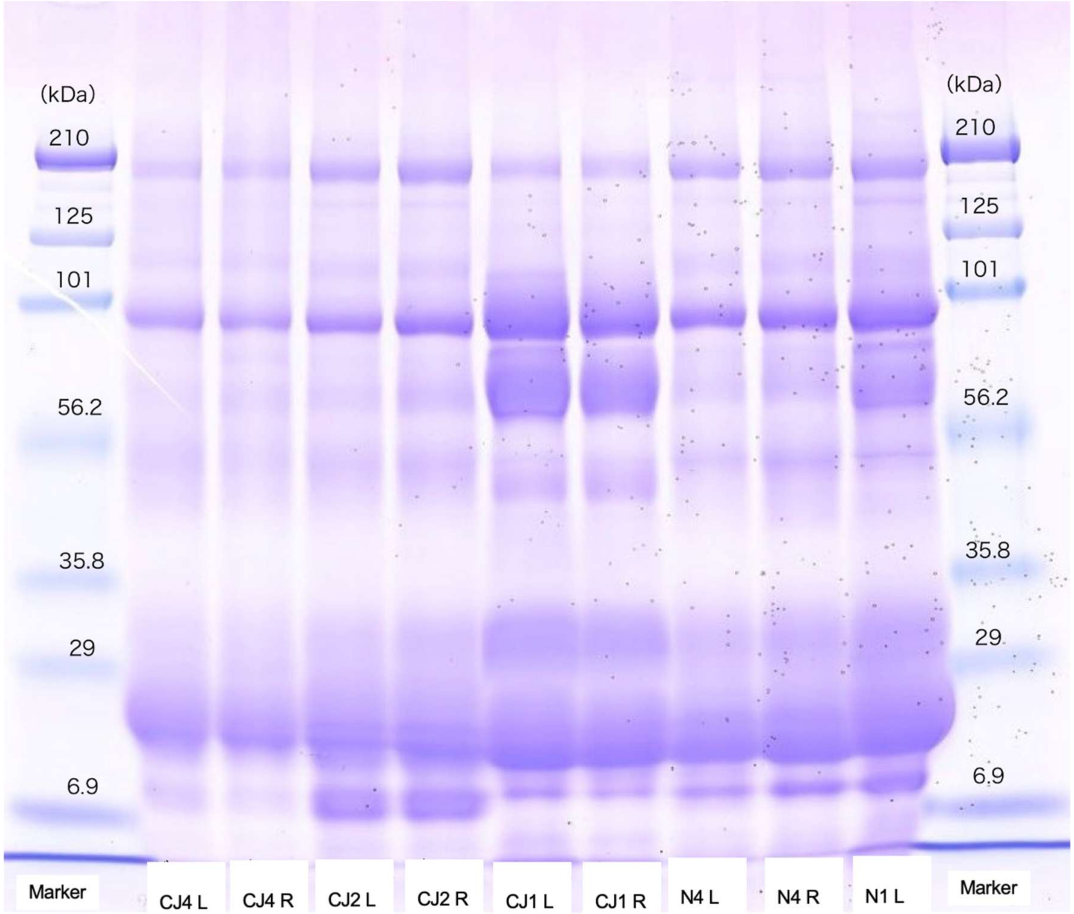

Figure S3. SDS–PAGE comparison of tear protein profiles between healthy cats and cats with conjunctivitis (run 1)

Figure S3. Sodium dodecyl sulfate–polyacrylamide gel electrophoresis (SDS–PAGE) profiles of tear protein samples obtained from healthy cats and cats with conjunctivitis. Sample identifiers are shown below each lane; "CJ" indicates cats affected by conjunctivitis, whereas "N" indicates healthy cats. The outermost lanes contain prestained molecular weight markers (BIO-RAD, Prestained SDS–PAGE Standards, Broad Range). To enable direct comparison between groups, tear samples from healthy and conjunctivitis cats were electrophoresed on the same gel under identical experimental conditions.

No protein bands uniquely present in the conjunctivitis group were observed when compared with the healthy cat group. However, in the healthy cat group, one to two faint high–molecular-weight bands (>210 kDa) were detected in samples from cats N1 and N4. The presence of faint high–molecular-weight bands was inconsistent across individuals and

was not detected in conjunctivitis samples, suggesting individual variation rather than disease-specific qualitative changes.

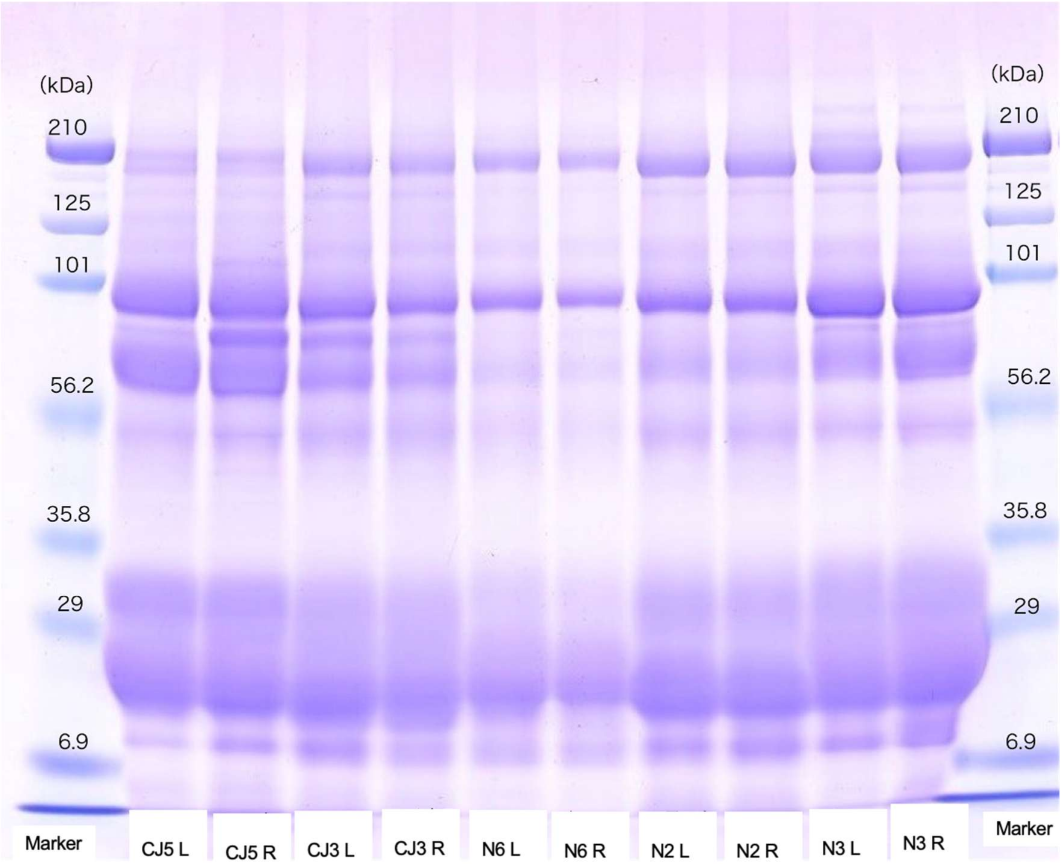

Figure S4. SDS–PAGE comparison of tear protein profiles between healthy cats and cats with conjunctivitis (run 2)

Figure S4. Sodium dodecyl sulfate–polyacrylamide gel electrophoresis (SDS–PAGE) profiles of tear protein samples obtained from healthy cats and cats with conjunctivitis. Sample identifiers are shown below each lane; "CJ" indicates cats affected by conjunctivitis, whereas "N" indicates healthy cats. The outermost lanes contain prestained molecular weight markers (BIO-RAD, Prestained SDS–PAGE Standards, Broad Range). To enable direct comparison between groups, tear samples from healthy and conjunctivitis cats were electrophoresed on the same gel under identical experimental conditions.

As in run 1, no protein bands uniquely present in the conjunctivitis group were observed. However, in the healthy cat group, one to two faint high–molecular-weight bands (>210 kDa) were detected in the sample from cat N3.

These high–molecular-weight bands were not consistently observed across individuals and were absent in conjunctivitis samples, suggesting individual variation rather than disease-specific qualitative changes.

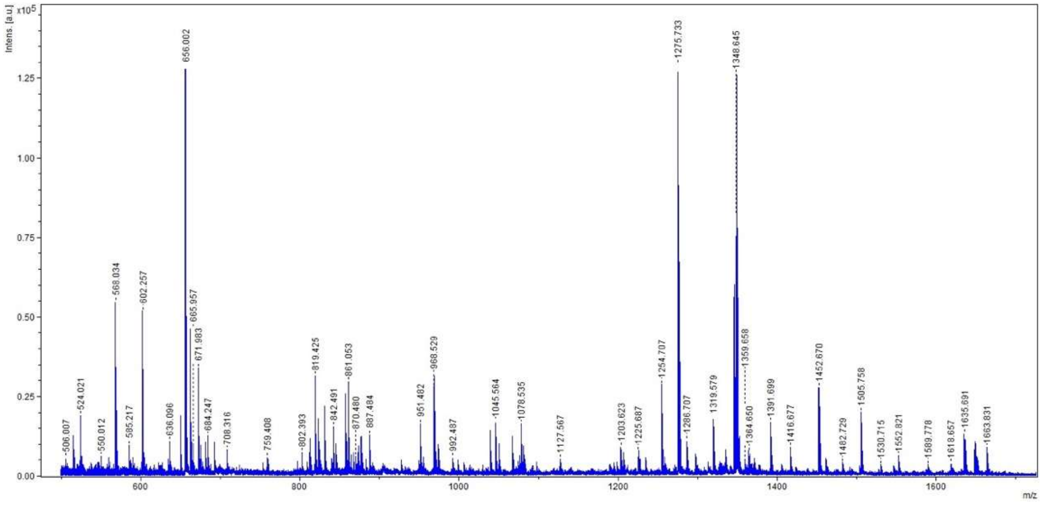

[−] **Detailed Results**

1. 23/74 matches (31%).
Acc. #: M3WGL8 **Uniprot ID:** M3WGL8_FELCA **Species:** FELCA **Name:** Lactoperoxidase
Organism: Felis catus OX=9685 **Gene:** LPO **Existence:** Predicted **Version:** 3
Index: 29163789 **MW:** 79949 Da **pI:** 8.8

m/z Submitted	MH+ Matched	Intensity	Delta ppm	Modifications	Start	End	Missed Cleavages	Sequence
819.3998	819.4472	100.0	-57.7		634	639	0	(K)QFQQIR(D)
831.4114	831.4512	100.0	-47.8		440	445	0	(K)WLPPYR(G)
857.3475	857.3797	100.0	-37.5		273	278	0	(K)C(Carbamidomethyl)MPFFR(A)
873.3543	873.3746	100.0	-23.3	1Oxidation	273	278	0	(K)C(Carbamidomethyl)MPFFR(A)
955.4866	955.5207	100.0	-35.7		505	513	0	(K)EGGIDPLVR(G)
968.5076	968.5312	100.0	-24.4		184	192	0	(R)NGFPLPPAR(E)
1039.5652	1039.5756	100.0	-9.95		148	157	1	(R)RNPALGAANR(A)
1050.4358	1050.4633	100.0	-26.2		139	147	0	(R)TITGDC(Carbamidomethyl)NNR(R)
1067.4559	1067.4575	100.0	-1.51		131	138	1	(K)C(Carbamidomethyl)DKDNPYR(T)
1076.5483	1076.5769	100.0	-26.5		528	536	1	(K)MITEELRGK(L)
1189.5647	1189.5848	100.0	-16.9		86	95	0	(R)NGQVWEESLK(R)
1206.5646	1206.5644	100.0	0.192		139	148	1	(R)TITGDC(Carbamidomethyl)NNRR(N)
1225.6574	1225.6800	100.0	-18.5		182	192	1	(R)TRNGFPLPPAR(E)
1319.5686	1319.5586	100.0	7.62		559	569	0	(R)DHGMPGYNSWR(S)
1345.6721	1345.6859	100.0	-10.2		86	96	1	(R)NGQVWEESLKR(L)
1348.5448	1348.6314	100.0	-64.2		465	476	0	(R)FGHTEVPSTMSR(L)
1364.6015	1364.6263	100.0	-18.2	1Oxidation	465	476	0	(R)FGHTEVPSTMSR(L)
1552.7931	1552.8264	100.0	-21.5		668	680	1	(R)LVC(Carbamidomethyl)DNTHITKVPR(H)
1635.6541	1635.6903	100.0	-22.2		557	569	1	(R)C(Carbamidomethyl)RDHGMPGYNSWR(S)
1635.6541	1635.8193	100.0	-101	1Oxidation	522	534	1	(K)LMNQNKMITEELR(G)
1651.6382	1651.6853	100.0	-28.5	1Oxidation	557	569	1	(R)C(Carbamidomethyl)RDHGMPGYNSWR(S)
1651.6382	1651.8142	100.0	-107	2Oxidation	522	534	1	(K)LMNQNKMITEELR(G)
1663.7930	1663.8261	100.0	-19.9		279	293	0	(R)AGFVC(Carbamidomethyl)PTPPYQSLAR(D)
1855.8554	1855.9086	100.0	-28.7		399	413	1	(R)LNPHWDGEKLYQEAR(K)
2766.2294	2766.5699	100.0	-123	1Oxidation	1	26	0	(-)MQALHLSVLLAFLTLLQAAALATNAR(A)

Figure S5. Mass spectrometric identification of lactoperoxidase from tryptic digest of Band 4

Figure S5. Representative mass spectrometric analysis of the tryptic digest obtained from Band 4 excised from the SDS–PAGE gel. The upper panel shows the acquired *m/z* spectrum of the tryptic peptides, whereas the lower panel highlights peaks that matched the theoretical peptide masses of lactoperoxidase in the database. The peptide matching rate

(Matches) was 31%, supporting the identification of lactoperoxidase as a constituent of the protein band.

Protein identification was based on peptide mass fingerprinting and database searching, and the matching score was considered sufficient for confident protein assignment under the applied analytical conditions.

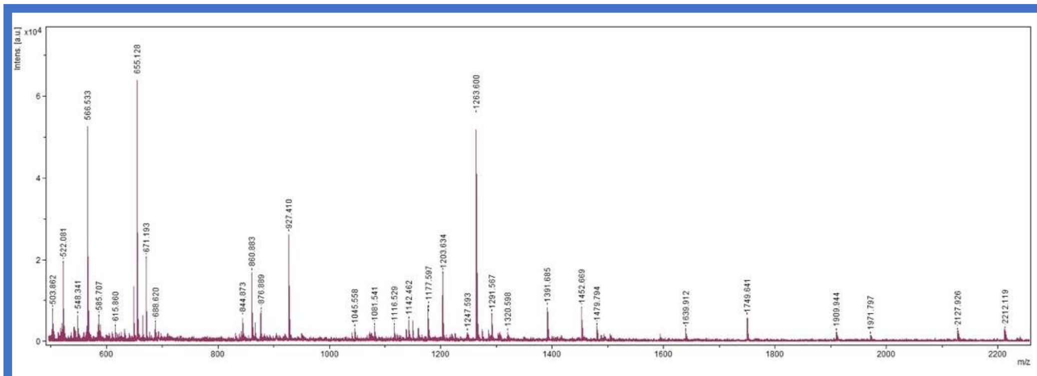

[–] **Detailed Results**

1. 14/56 matches (25%).
Acc. #: A0A2I2U7Y0 **Uniprot ID:** A0A2I2U7Y0_FELCA **Species:** FELCA **Name:** Albumin
Organism: Felis catus OX=9685 **Gene:** ALB **Existence:** Predicted **Version:** 3
Index: 29176371 **MW:** 69706 Da **pI:** 5.3

m/z Submitted	MH+ Matched	Intensity	Delta ppm	Modifications	Start	End	Missed Cleavages	Sequence
927.4096	927.4934	100.0	-90.4		172	178	0	(K)YLYEIAR(R)
1045.5579	1045.5313	100.0	25.4		99	107	0	(K)SLHELFGDK(L)
1116.5294	1116.5102	100.0	17.2		321	329	0	(K)SHC(Carbamidomethyl)ISEVER(D)
1137.5214	1137.4881	100.0	29.2		599	608	0	(K)EAC(Carbamidomethyl)FAEEGPK(L)
1149.6378	1149.6150	100.0	19.8		76	85	0	(K)LVNEVTEFAK(G)
1159.5533	1159.4619	100.0	78.8		471	479	0	(K)C(Carbamidomethyl)C(Carbamidomethyl)THPEAER(L)
1177.5973	1177.5709	100.0	22.4		35	44	0	(R)EAHQSEIAHR(F)
1263.6003	1263.5753	100.0	19.8		45	54	0	(R)FNDLGEEHFR(G)
1479.7939	1479.7954	100.0	-1.07		432	444	0	(K)LGEYGFQNALLVR(Y)
1639.9116	1639.9377	100.0	-15.9		448	462	1	(K)KVPQVSTPTLVEVSR(S)
1749.6409	1749.6625	100.0	-12.4		278	291	0	(K)EC(Carbamidomethyl)C(Carbamidomethyl)HGDLLEC(Carbamidomethyl)ADDR(A)
1909.9438	1909.9477	100.0	-2.03		519	534	0	(R)RPC(Carbamidomethyl)FSALQVDETYVPK(E)
1971.7974	1971.7881	100.0	4.71		592	608	1	(K)C(Carbamidomethyl)C(Carbamidomethyl)AAEDKEAC(Carbamidomethyl)FAEEGPK(L)
2127.9256	2127.9005	100.0	11.8		275	291	1	(K)IHKEC(Carbamidomethyl)C(Carbamidomethyl)HGDLLEC(Carbamidomethyl)ADDR(A)
2127.9256	2127.9508	100.0	-11.8		197	215	1	(K)GVFTEC(Carbamidomethyl)C(Carbamidomethyl)EAADKAAC(Carbamidomethyl)LTPK(V)

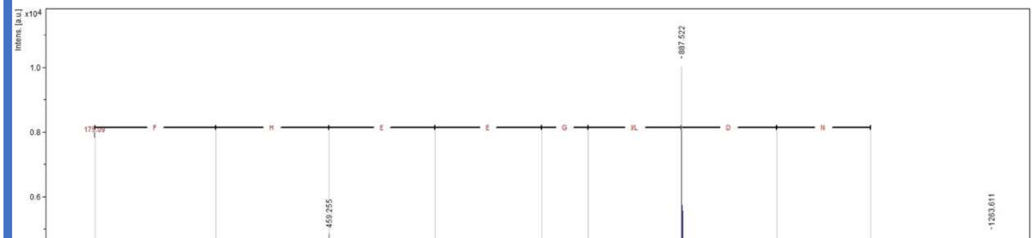

Figure S6. Mass spectrometric identification of albumin from tryptic digest of Band 6

Figure S6. Representative mass spectrometric analysis of the tryptic digest obtained from Band 6 excised from the SDS–PAGE gel. The upper panel shows the acquired *m/z* spectrum of the tryptic peptides, and the middle panel highlights peaks that matched the theoretical peptide masses of albumin in the database. The peptide matching rate (Matches) was 25%, supporting the identification of albumin as a major component of the protein band. In

addition, tandem mass spectrometric analysis of the ion at *m/z* 1263.6 was performed using LIFT mode (lower panel), and the resulting fragment ion spectrum was consistent with the corresponding amino acid sequence in the MS-FIT database, providing further confirmation of albumin identification.

Protein identification was based on peptide mass fingerprinting combined with tandem mass spectrometric validation.

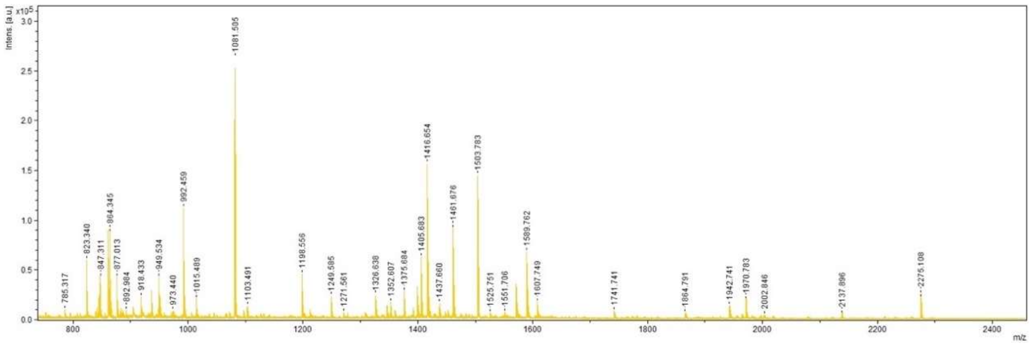

1. 36/65 matches (55%).
Acc. #: M3WKU0 **Uniprot ID:** M3WKU0_FELCA **Species:** FELCA **Name:** Lactotransferrin
Organism: Felis catus OX=9685 **Gene:** LTF **Existence:** Inferred from homology **Version:** 3
Index: 29178765 **MW:** 82056 Da **pI:** 8.5

m/z Submitted	MH+ Matched	Intensity	Delta ppm	Modifications	Start	End	Missed Cleavages	Sequence
785.3172	785.3723	100.0	-70.1		263	268	0	(K)EC(Carbamidomethyl)HLAR(I)
823.3397	823.3879	100.0	-58.6		41	46	0	(K)FQQNMR(E)
839.3344	839.3828	100.0	-57.7	1Oxidation	41	46	0	(K)FQQNMR(E)
861.3861	861.4141	100.0	-32.5		502	508	0	(K)FGEFFSK(S)
864.3453	864.3781	100.0	-38.0		377	382	0	(K)C(Carbamidomethyl)QQWSR(A)
878.3921	878.4730	100.0	-92.2		321	328	0	(K)DSALGFLR(V)
887.3696	887.4152	100.0	-51.4		133	140	0	(K)SC(Carbamidomethyl)HTGLGR(S)
918.4330	918.4614	100.0	-30.9		364	371	0	(R)VAWC(Carbamidomethyl)AVGR(E)
936.4761	936.5010	100.0	-26.6		611	619	0	(R)APNHGVVSR(E)
949.5339	949.4408	100.0	98.1		191	199	0	(R)LC(Carbamidomethyl)AGTGENK(C)
949.5339	949.5578	100.0	-25.1		269	277	0	(R)IPSHAVVAR(S)
951.4091	951.4353	100.0	-27.5		184	190	0	(K)EYPNLC(Carbamidomethyl)R(L)
992.4592	992.4731	100.0	-14.0		376	382	1	(R)KC(Carbamidomethyl)QQWSR(A)
1015.4886	1015.5102	100.0	-21.3		132	140	1	(R)KSC(Carbamidomethyl)HTGLGR(S)
1081.5051	1081.5102	100.0	-4.64		542	550	0	(R)YFGYTGAFR(C)
1198.5556	1198.5633	100.0	-6.44		47	57	0	(R)EVGGPHVSC(Carbamidomethyl)TR(K)
1212.5623	1212.5612	100.0	0.852		38	46	1	(K)C(Carbamidomethyl)TKFQQNMR(E)
1249.5849	1249.5888	100.0	-3.10		206	216	0	(R)EPYFGYAGAFK(C)
1308.6388	1308.6655	100.0	-20.4		611	622	1	(R)APNHGVVSREDK(A)
1326.6383	1326.6583	100.0	-15.0		47	58	1	(R)EVGGPHVSC(Carbamidomethyl)TRK(N)
1346.6601	1346.6845	100.0	-18.1		59	69	1	(K)NSRQEC(Carbamidomethyl)IQAIK(A)
1352.6067	1352.6151	100.0	-6.17		27	37	0	(R)WC(Carbamidomethyl)TISQAEETK(C)
1375.6836	1375.7077	100.0	-17.5		120	131	0	(K)GTNFQLNQLQGR(K)
1405.6830	1405.7070	100.0	-17.1		460	471	1	(R)KSDADITWNSLR(G)
1416.6543	1416.6689	100.0	-10.3		217	229	0	(K)C(Carbamidomethyl)LHEGGGDVAFVR(H)
1461.6764	1461.6903	100.0	-9.54		364	375	1	(R)VAWC(Carbamidomethyl)AVGREEER(K)
1503.7833	1503.8027	100.0	-12.9		120	132	1	(K)GTNFQLNQLQGRK(S)
1503.7833	1503.8027	100.0	-12.9		119	131	1	(K)KGTNFQLNQLQGR(K)
1551.7055	1551.7472	100.0	-26.8		657	669	0	(K)NLLFNDNTEC(Carbamidomethyl)LAK(L)
1570.7847	1570.8159	100.0	-19.8		256	268	1	(R)KPVDAFKEC(Carbamidomethyl)HLAR(I)
1607.7486	1607.7846	100.0	-22.4		25	37	1	(R)VRWC(Carbamidomethyl)TISQAEETK(C)
1741.7405	1741.7884	100.0	-27.5		27	40	1	(R)WC(Carbamidomethyl)TISQAEETKC(Carbamidomethyl)TK(F)
1864.7912	1864.8672	100.0	-40.8		564	581	0	(K)DSTVLQNTNGGSSEAWAK(D)
1970.7832	1970.8524	100.0	-35.1		200	216	1	(K)C(Carbamidomethyl)AC(Carbamidomethyl)SSREPYFGYAGAFK(C)
2002.8463	2002.9334	100.0	-43.5		41	57	1	(K)FQQNMREVGGPHVSC(Carbamidomethyl)TR(K)
2652.1811	2652.2900	100.0	-41.1		556	581	1	(K)AGDVAFVKDSTVLQNTNGGSSEAWAK(D)

Figure S7. Mass spectrometric identification of lactotransferrin from tryptic digest of Band 5

Figure S7. Representative mass spectrometric analysis of the tryptic digest obtained from Band 5 excised from the SDS–PAGE gel. The upper panel shows the acquired *m/z* spectrum of the tryptic peptides, whereas the lower panel highlights peaks that matched the

theoretical peptide masses of lactotransferrin in the database. The peptide matching rate (Matches) exceeded 30%, supporting the identification of lactotransferrin as a major component of the protein band.
Protein identification was based on peptide mass fingerprinting and database matching under the applied analytical conditions.

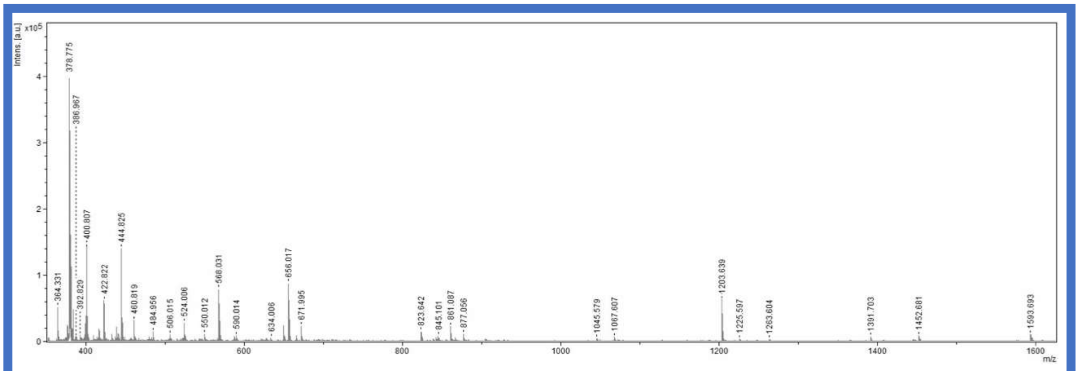

1. 4/71 matches (5%).
Acc. #: A0A291NGR8 **Uniprot ID:** A0A291NGR8_FELCA **Species:** FELCA **Name:** IgA constant region (Fragment)
Organism: Felis catus OX=9685 **Existence:** Evidence at transcript level **Version:** 1
Index: 29187842 **MW:** 42040 Da **pI:** 5.7

m/z Submitted	MH+ Matched	Intensity	Delta ppm	Modifications	Start	End	Missed Cleavages	Sequence
1067.6071	1067.5844	100.0	21.3		200	208	0	(K)EAIQKPPER(D)
1203.6387	1203.6117	100.0	22.5		296	305	0	(R)WLQGSQELSR(E)
1263.6041	1263.5562	100.0	37.9		1	10	0	(-)EDTAIYYC(Carbamidomethyl)TK(T)
1593.6927	1593.6784	100.0	8.96		144	157	0	(R)ADPPPPC(Carbamidomethyl)SPC(Carbamidomethyl)NEPR(L)

Num Unmatched Masses: **67**
Search for disulfide linked peptides.
Do a non-specific cleavage search.
Search for another component.

The matched peptides cover **11.2%** (43/385AA's) of the protein.
Coverage Map for This Hit (MS-Digest index #): 29187842

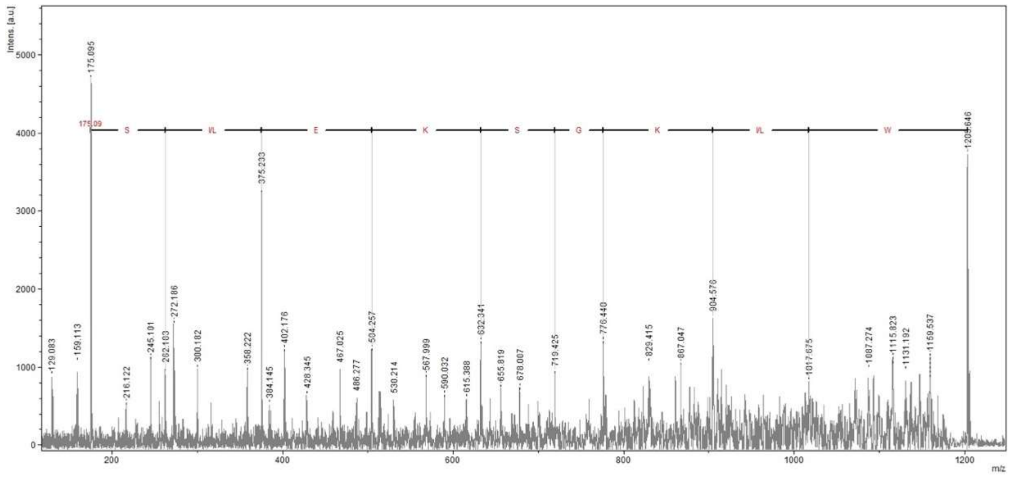

Figure S8. Mass spectrometric identification of the IgA constant region from tryptic digest of Band 7

Figure S8. Representative mass spectrometric analysis of the tryptic digest obtained from Band 7 excised from the SDS–PAGE gel. The upper panel shows the acquired *m/z* spectrum of the tryptic peptides, and the middle panel highlights peaks that matched the theoretical peptide masses of the immunoglobulin A (IgA) constant region in the database. In addition,

tandem mass spectrometric analysis of the ion at *m/z* 1203.6 was performed using LIFT mode (lower panel), and the resulting fragment ion spectrum was consistent with the corresponding amino acid sequence in the MS-FIT database, providing further confirmation of IgA constant region identification.

Protein identification was achieved by peptide mass fingerprinting combined with tandem mass spectrometric validation.

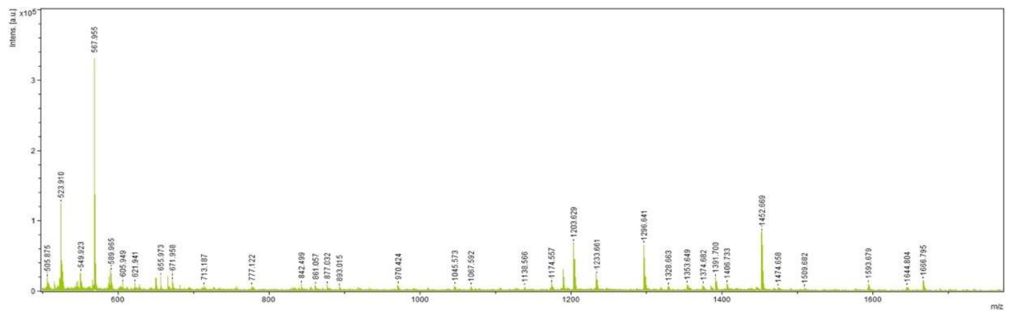

[–] **Detailed Results**

1. 7/67 matches (10%).
Acc. #: M3VXE7 **Uniprot ID:** M3VXE7_FELCA **Species:** FELCA **Name:** Selenium binding protein 1
Organism: Felis catus OX=9685 **Gene:** SELENBP1 **Existence:** Inferred from homology **Version:** 3
Index: 29141947 **MW:** 52572 Da **pI:** 5.9

m/z Submitted	MH+ Matched	Intensity	Delta ppm	Modifications	Start	End	Missed Cleavages	Sequence
1233.6610	1233.6474	100.0	11.1		104	114	0	(R)IYVVDVGSEPR(A)
1296.6409	1296.6266	100.0	11.1		53	62	0	(K)SPHYC(Carbamidomethyl)QVIHR(L)
1374.6824	1374.6761	100.0	4.60		334	344	0	(R)QYDISDPQRPR(L)
1452.6691	1452.7371	100.0	-46.9	1Oxidation	92	103	1	(R)TKLMLPC(Carbamidomethyl)LMSSR(I)
1468.7360	1468.7321	100.0	2.67	2Oxidation	92	103	1	(R)TKLMLPC(Carbamidomethyl)LMSSR(I)
1468.7360	1468.7505	100.0	-9.87		24	34	0	(R)EEIIYLPC(Carbamidomethyl)IYR(N)
1917.9302	1917.9440	100.0	-7.20		35	52	0	(R)NTGIEAPDYLATVDVDPK(S)
2324.9053	2324.9295	100.0	-10.4		70	89	0	(K)DELHHSGWNTC(Carbamidomethyl)SSC(Carbamidomethyl)FGDSTK(S)

Num Unmatched Masses: **60**
Search for disulfide linked peptides.
Do a non-specific cleavage search.
Search for another component.

The matched peptides cover **19.7%** (93/472AA's) of the protein.
Coverage Map for This Hit (MS-Digest index #): 29141947

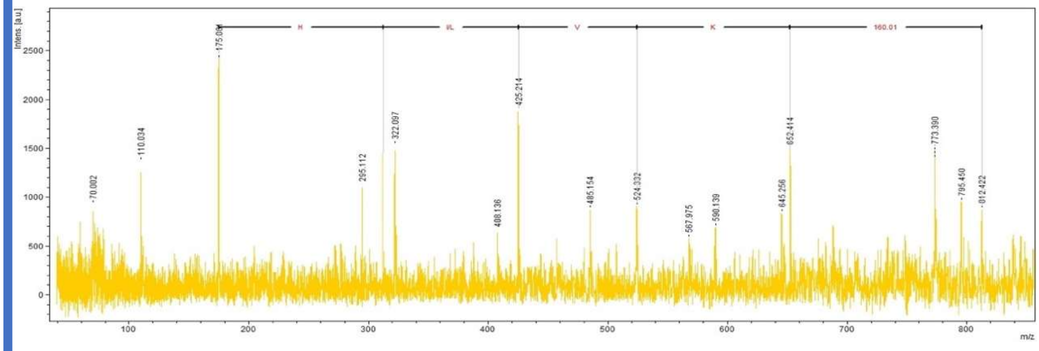

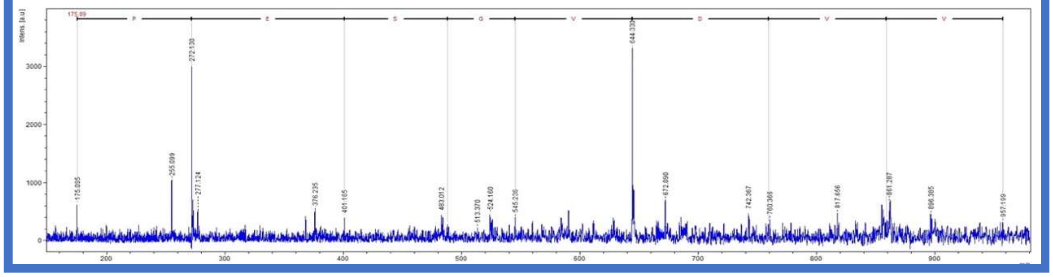

Figure S9. Mass spectrometric identification of selenium-binding protein 1 from tryptic digest of Band 8

Figure S9. Representative mass spectrometric analysis of the tryptic digest obtained from

Band 8 excised from the SDS–PAGE gel. The upper panel shows the acquired *m/z* spectrum of the tryptic peptides, whereas the middle panel highlights peaks that matched the theoretical peptide masses of selenium-binding protein 1 in the database. Tandem mass spectrometric analyses of the ions at *m/z* 1233.6 and 1296.6 were subsequently performed using LIFT mode (two lower panels), and the resulting fragment ion spectra were consistent with the corresponding amino acid sequences in the MS-FIT database, thereby providing further confirmation of selenium-binding protein 1 identification.
Protein identification was based on peptide mass fingerprinting combined with tandem mass spectrometric validation.

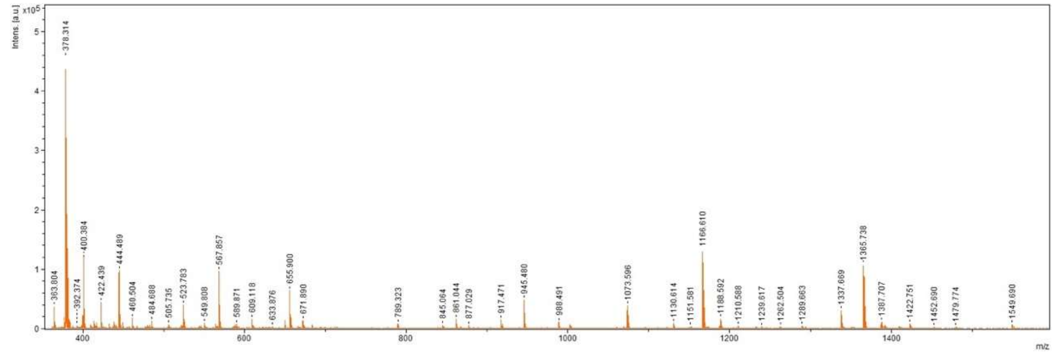

1. 12/98 matches (12%).
Acc. #: E5D2Z5 **Uniprot ID:** E5D2Z5_FELCA **Species:** FELCA **Name:** Fel d 7 allergen
Organism: Felis catus OX=9685 **Gene:** LOC100533977 **Existence:** Evidence at transcript level **Version:** 1
Index: 28292262 **MW:** 20185 Da **pI:** 4.9

m/z Submitted	MH+ Matched	Intensity	Delta ppm	Modifications	Start	End	Missed Cleavages	Sequence
789.3228	789.3777	100.0	-69.6		95	101	0	(K)YTTYGGK(R)
917.4713	917.4727	100.0	-1.58		94	101	1	(K)KYTTYGGK(R)
945.4800	945.4789	100.0	1.24		95	102	1	(K)YTTYGGKR(V)
1166.6103	1166.5840	100.0	22.5		156	165	0	(K)GFNQEIFSPK(Q)
1337.6688	1337.6518	100.0	12.7		39	49	1	(K)AMITDRETSWK(K)
1365.7380	1365.7161	100.0	16.0		154	165	1	(R)AKGFNQEIFSPK(Q)
1549.6904	1549.6765	100.0	8.95		137	149	0	(R)DPESNEEALENFR(E)
1974.9602	1974.9516	100.0	4.38		133	149	1	(K)LVGRDPESNEEALENFR(E)
2132.8865	2132.8801	100.0	3.03		113	129	0	(K)DHYIFYC(Carbamidomethyl)EGEMQGEQAR(M)
2148.8824	2148.8750	100.0	3.44	1Oxidation	113	129	0	(K)DHYIFYC(Carbamidomethyl)EGEMQGEQAR(M)
3290.4947	3290.5133	100.0	-5.66		103	129	1	(R)VVYIEPTEVKDHYIFYC(Carbamidomethyl)EGEMQGEQAR(M)
3306.4933	3306.5082	100.0	-4.52	1Oxidation	103	129	1	(R)VVYIEPTEVKDHYIFYC(Carbamidomethyl)EGEMQGEQAR(M)

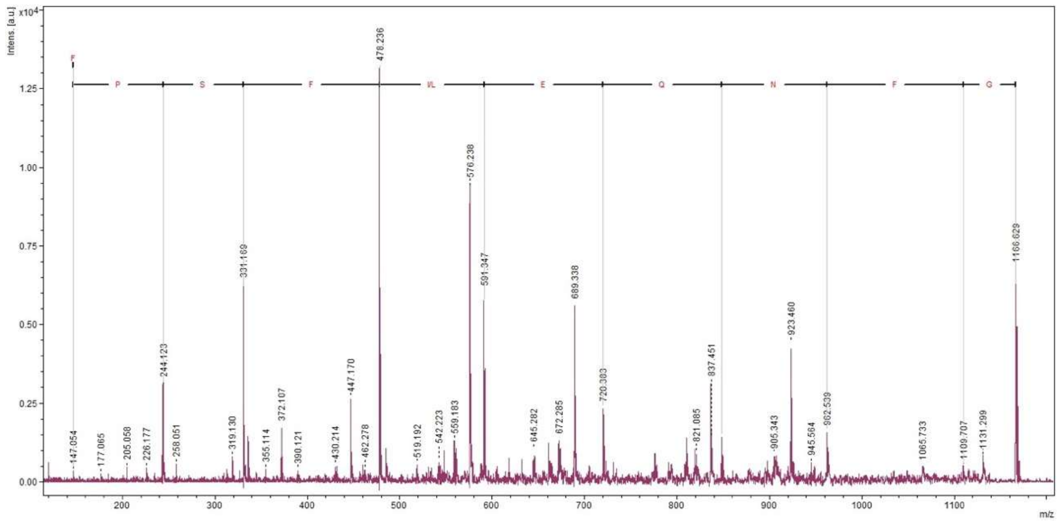

Figure S10. Mass spectrometric identification of Fel d 7 allergen from tryptic digest of Band 13

Figure S10. Representative mass spectrometric analysis of the tryptic digest obtained from Band 13 excised from the SDS–PAGE gel. The upper panel shows the acquired *m/z*

spectrum of the tryptic peptides, whereas the middle panel highlights peaks that matched the theoretical peptide masses of Fel d 7 allergen in the database. Tandem mass spectrometric analysis of the ion at *m/z* 1166.6 was subsequently performed using LIFT mode (lower panel), and the resulting fragment ion spectrum was consistent with the corresponding amino acid sequence in the MS-FIT database, thereby providing further confirmation of Fel d 7 allergen identification.

Fel d 7 allergen is a known feline allergen-related protein, and its detection in tear samples may reflect immune-related components of the ocular surface environment.

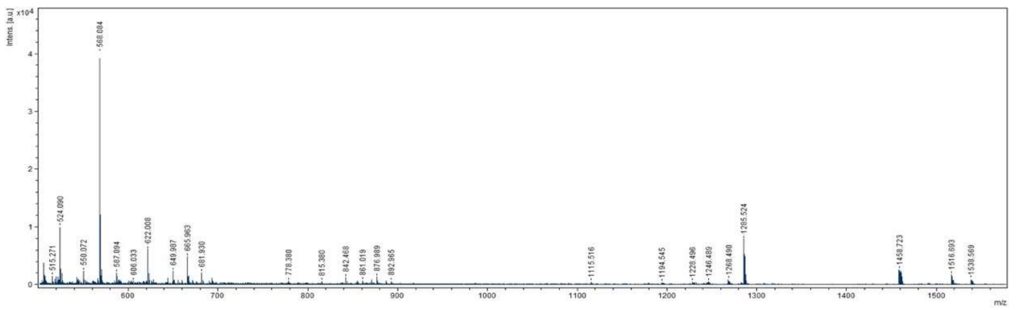

[–] **Detailed Results**

1. 4/58 matches (6%).
Acc. #: M3W0R2 **Uniprot ID:** M3W0R2_FELCA **Species:** FELCA **Name:** SRCR domain-containing protein
Organism: Felis catus OX=9685 **Existence:** Predicted **Version:** 4
Index: 29141996 **MW:** 24057 Da **pI:** 6.5

m/z Submitted	MH+ Matched	Intensity	Delta ppm	Modifications	Start	End	Missed Cleavages	Sequence
1231.5594	1231.5960	100.0	-29.7		53	63	1	(R)LVNGGDRC(Carbamidomethyl)QGR(V)
1285.5245	1285.5630	100.0	-30.0		120	129	0	(R)C(Carbamidomethyl)SGQESYLWR(C)
1458.7227	1458.7700	100.0	-32.4		106	119	0	(R)FGQGSGPIVLDNVR(C)
1516.6930	1516.7325	100.0	-26.0		91	105	0	(R)QLGC(Carbamidomethyl)GWAVSAPGSAR(F)

Num Unmatched Masses: **54**
Search for disulfide linked peptides.
Do a non-specific cleavage search.
Search for another component.

The matched peptides cover **22.6%** (50/221AA's) of the protein.
Coverage Map for This Hit (MS-Digest index #): 29141996

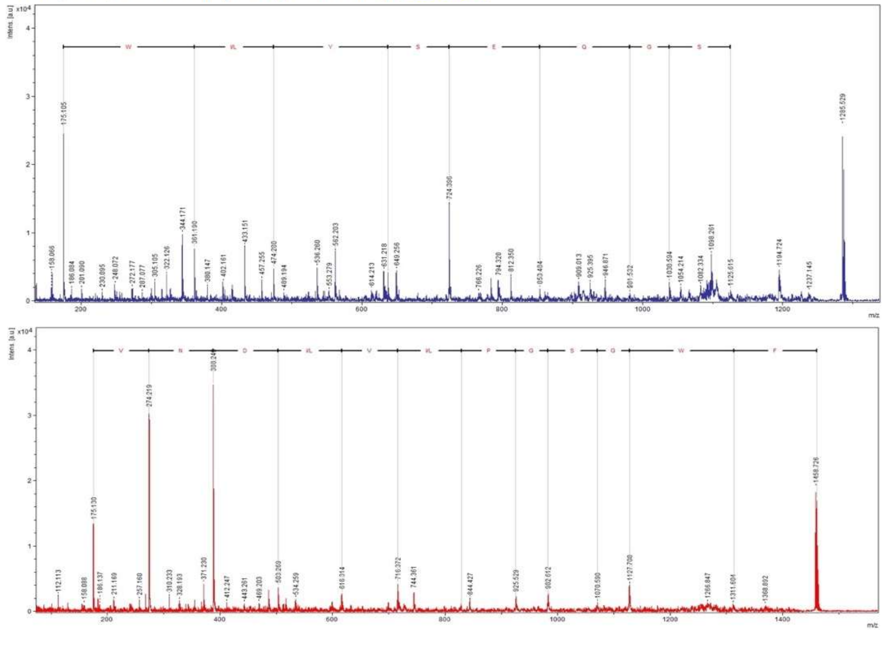

Figure S11. Mass spectrometric identification of an SRCR domain–containing protein from tryptic digest of Band 14

Figure S11. Representative mass spectrometric analysis of the tryptic digest obtained from

Band 14 excised from the SDS–PAGE gel. The upper panel shows the acquired *m/z* spectrum of the tryptic peptides, whereas the middle panel highlights peaks that matched the theoretical peptide masses of an SRCR (scavenger receptor cysteine-rich) domain–containing protein in the database. Tandem mass spectrometric analyses of the ions at *m/z* 1285.5 and 1458.7 were subsequently performed using LIFT mode (two lower panels), and the resulting fragment ion spectra were consistent with the corresponding amino acid sequences in the MS-FIT database, thereby providing further confirmation of SRCR domain–containing protein identification.

SRCR domain–containing proteins are associated with innate immune functions, and their detection in tear samples may reflect immune-related components of the ocular surface, although functional implications were not addressed in this study.

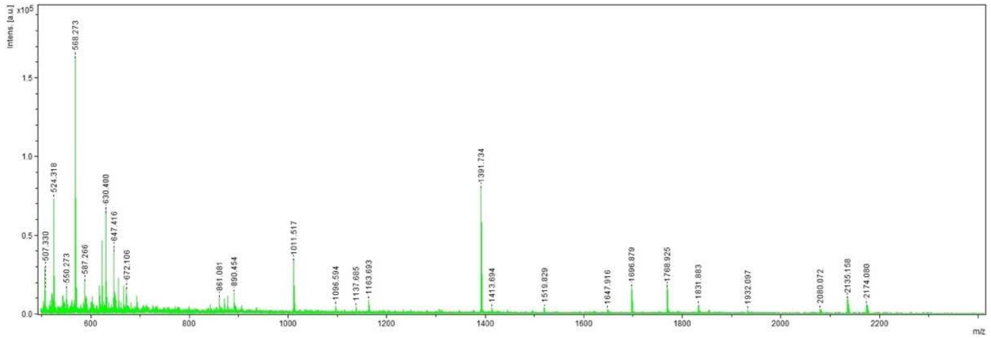

[–] **Detailed Results**

1. 16/52 matches (30%).
Acc. #: M3W7B1 **Uniprot ID:** M3W7B1_FELCA **Species:** FELCA **Name:** Coiled-coil domain containing 146
Organism: Felis catus OX=9685 **Gene:** CCDC146 **Existence:** Predicted **Version:** 2
Index: 29162850 **MW:** 112768 Da **pI:** 7.3

m/z Submitted	MH+ Matched	Intensity	Delta ppm	Modifications	Start	End	Missed Cleavages	Sequence
504.3193	504.2664	100.0	105		727	730	0	(R)DLEK(Q)
504.3193	504.2664	100.0	105		467	470	0	(K)IDEK(E)
504.3193	504.2776	100.0	82.7		785	788	0	(R)LTDR(L)
507.3303	507.2595	100.0	140		789	792	0	(R)LC(Carbamidomethyl)SK(T)
518.3252	518.2821	100.0	83.2		245	248	0	(K)EIEK(T)
522.3016	522.2922	100.0	17.9		476	479	0	(K)DFLK(A)
526.3281	526.2984	100.0	56.4		409	412	0	(R)ELHK(E)
543.2963	543.2885	100.0	14.3	1Gln->pyro-Glu	355	358	1	(K)QREK(E)
543.2963	543.3249	100.0	-52.7		60	64	0	(K)LPGTR(M)
561.3100	561.2879	100.0	39.4		294	298	0	(K)EVEGK(R)
561.3100	561.2991	100.0	19.3		357	360	1	(R)EKER(D)
590.2488	590.3330	100.0	-143		596	600	0	(K)LQAMK(E)
591.2926	591.3171	100.0	-41.3		65	69	0	(R)MAELK(A)
603.2747	603.3348	100.0	-99.7		289	293	0	(K)EDVIK(E)
630.4002	630.3821	100.0	28.6		126	130	0	(R)EQLLK(Y)
630.4002	630.4185	100.0	-29.1		194	198	1	(K)KLEIK(N)
636.2672	636.3021	100.0	-54.9		189	193	0	(K)ETMQK(K)
636.2672	636.3385	100.0	-112	1Oxidation	462	466	0	(R)MTQIK(I)
890.4537	890.5380	100.0	-94.6	1Oxidation	367	373	1	(R)KMELLLK(V)
1011.5168	1011.6197	100.0	-102		856	864	1	(R)IEKGLPLNK(D)
1137.6850	1137.6487	100.0	31.9		691	699	1	(K)QRQIHVTQK(L)
1413.6936	1413.6757	100.0	12.7		131	141	1	(K)YQNEYNAVKER(E)

Num Unmatched Masses: **36**
Search for disulfide linked peptides.
Do a non-specific cleavage search.
Search for another component.

The matched peptides cover **12.0%** (115/956AA's) of the protein.
Coverage Map for This Hit (MS-Digest index #): 29162850

Figure S12. Mass spectrometric identification of coiled-coil domain–containing protein 146 from tryptic digest of Band 12

Figure S12. Representative mass spectrometric analysis of the tryptic digest obtained from Band 12 excised from the SDS–PAGE gel. The upper panel shows the acquired *m/z* spectrum of the tryptic peptides, whereas the lower panel highlights peaks that matched the theoretical peptide masses of coiled-coil domain–containing protein 146 in the database.

The peptide matching rate (Matches) exceeded 30%, supporting the identification of coiled-coil domain–containing protein 146 as a constituent of the protein band.
The biological function of coiled-coil domain–containing protein 146 in tear fluid remains unclear, and its detection warrants further investigation.

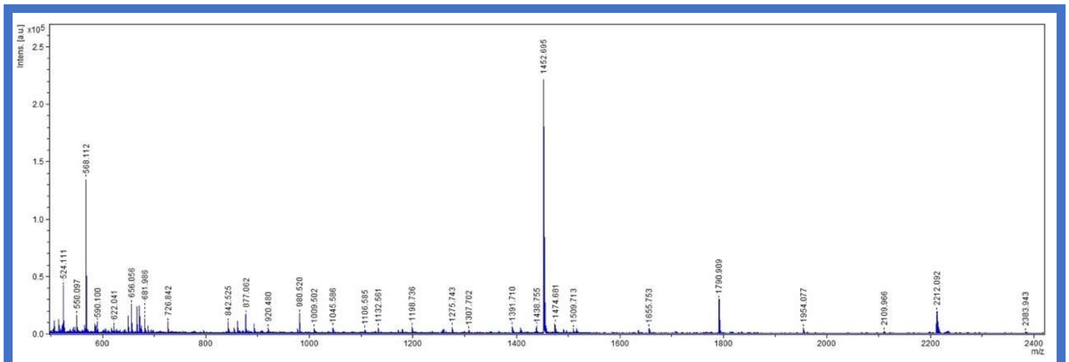

[-] **Detailed Results**

1. 7/66 matches (10%).
Acc. #: M3WRZ7 **Uniprot ID:** M3WRZ7_FELCA **Species:** FELCA **Name:** Uncharacterized protein
Organism: Felis catus OX=9685 **Gene:** ACTB **Existence:** Inferred from homology **Version:** 3
Index: 29148339 **MW:** 41737 Da **pI:** 5.3

m/z Submitted	MH+ Matched	Intensity	Delta ppm	Modifications	Start	End	Missed Cleavages	Sequence
976.4701	976.4483	100.0	22.3		19	28	0	(K)AGFAGDDAPR(A)
1132.5609	1132.5269	100.0	30.0		197	206	0	(R)GYSFTTTAER(E)
1198.7359	1198.7055	100.0	25.3		29	39	0	(R)AVFPSIVGRPR(H)
1516.7474	1516.7027	100.0	29.5		360	372	0	(K)QEYDESGPSIVHR(K)
1790.9085	1790.8919	100.0	9.27		239	254	0	(K)SYELPDGQVITIGNER(F)
1954.0769	1954.0644	100.0	6.42		96	113	0	(R)VAPEEHPVLLTEAPLNPK(A)
2215.0382	2215.0700	100.0	-14.3		292	312	0	(K)DLYANTVLSGGTTMYPGIADR(M)

Num Unmatched Masses: **59**
Search for disulfide linked peptides.
Do a non-specific cleavage search.
Search for another component.

The matched peptides cover **26.4%** (99/375AA's) of the protein.
Coverage Map for This Hit (MS-Digest index #): 29148339

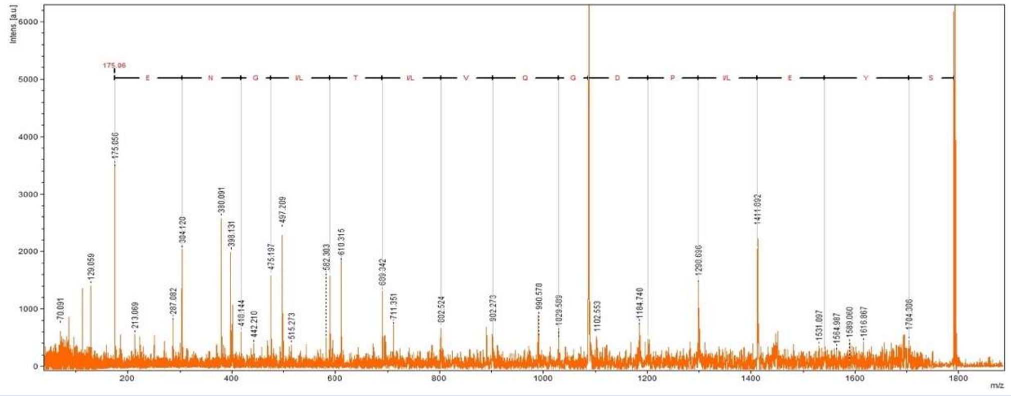

Figure S13. Mass spectrometric identification of an uncharacterized protein from tryptic digest of Band 9

Figure S13. Representative mass spectrometric analysis of the tryptic digest obtained from

Band 9 excised from the SDS–PAGE gel. The upper panel shows the acquired *m/z* spectrum of the tryptic peptides, whereas the middle panel highlights peaks that matched the theoretical peptide masses of an uncharacterized protein in the database. Tandem mass spectrometric analysis of the ion at *m/z* 1790.9 was subsequently performed using LIFT mode (lower panel), and the resulting fragment ion spectrum was consistent with the corresponding amino acid sequence in the MS-FIT database, thereby supporting the identification of this protein as an uncharacterized protein.

The biological function of this uncharacterized protein in feline tear fluid remains unknown, and its potential relevance to ocular surface physiology or disease requires further investigation.
