# Supplementary figures and images for: A Pilot Proteomic Analysis of Tear Fluid in Domestic Cats with and Without Conjunctivitis Using MALDI–TOF/TOF Mass Spectrometry
Source: Animals (Basel). 2026 Mar 13;16(6):912. doi: 10.3390/ani16060912 (PMC13023246; doi:10.3390/ani16060912)

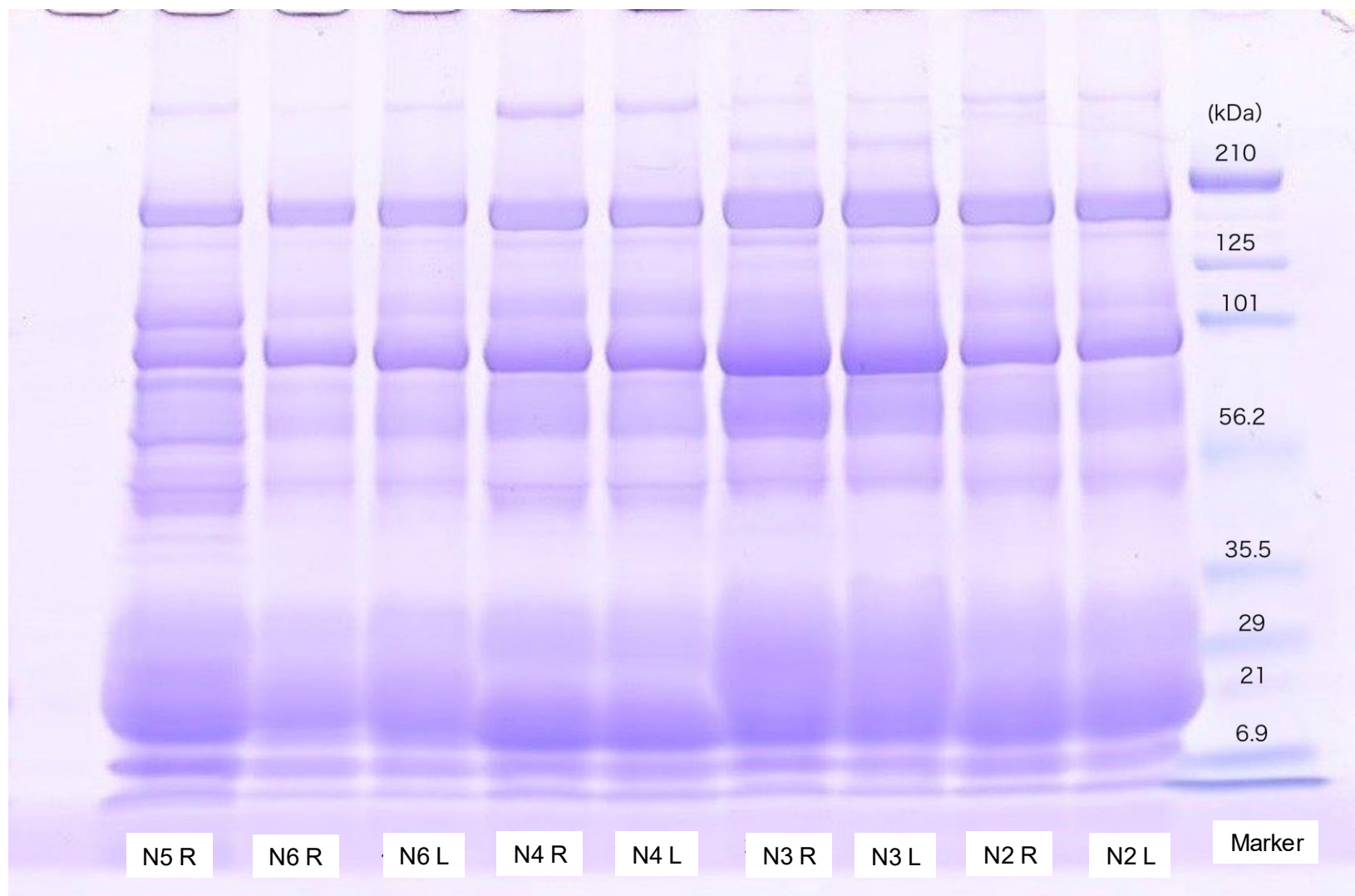



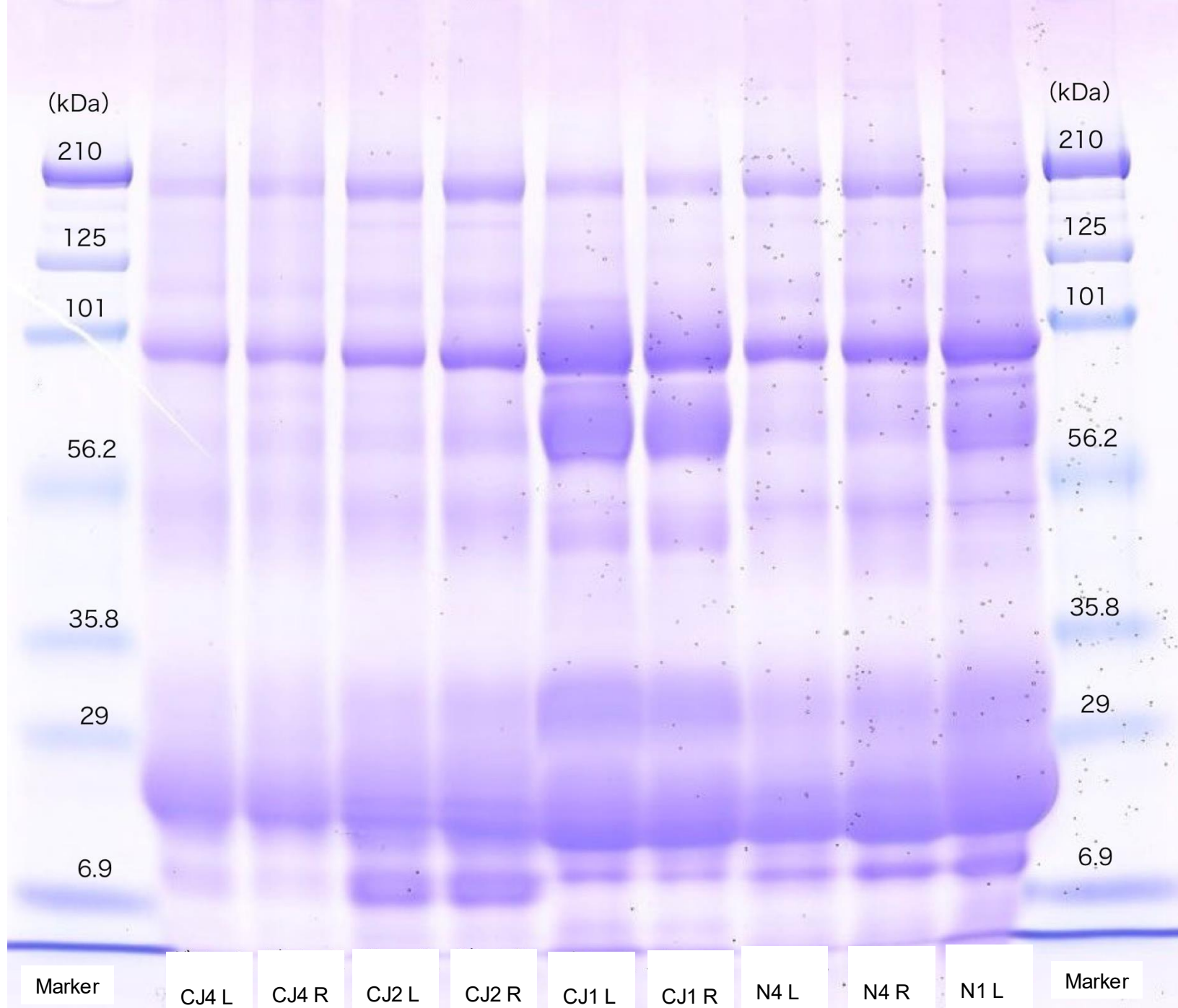

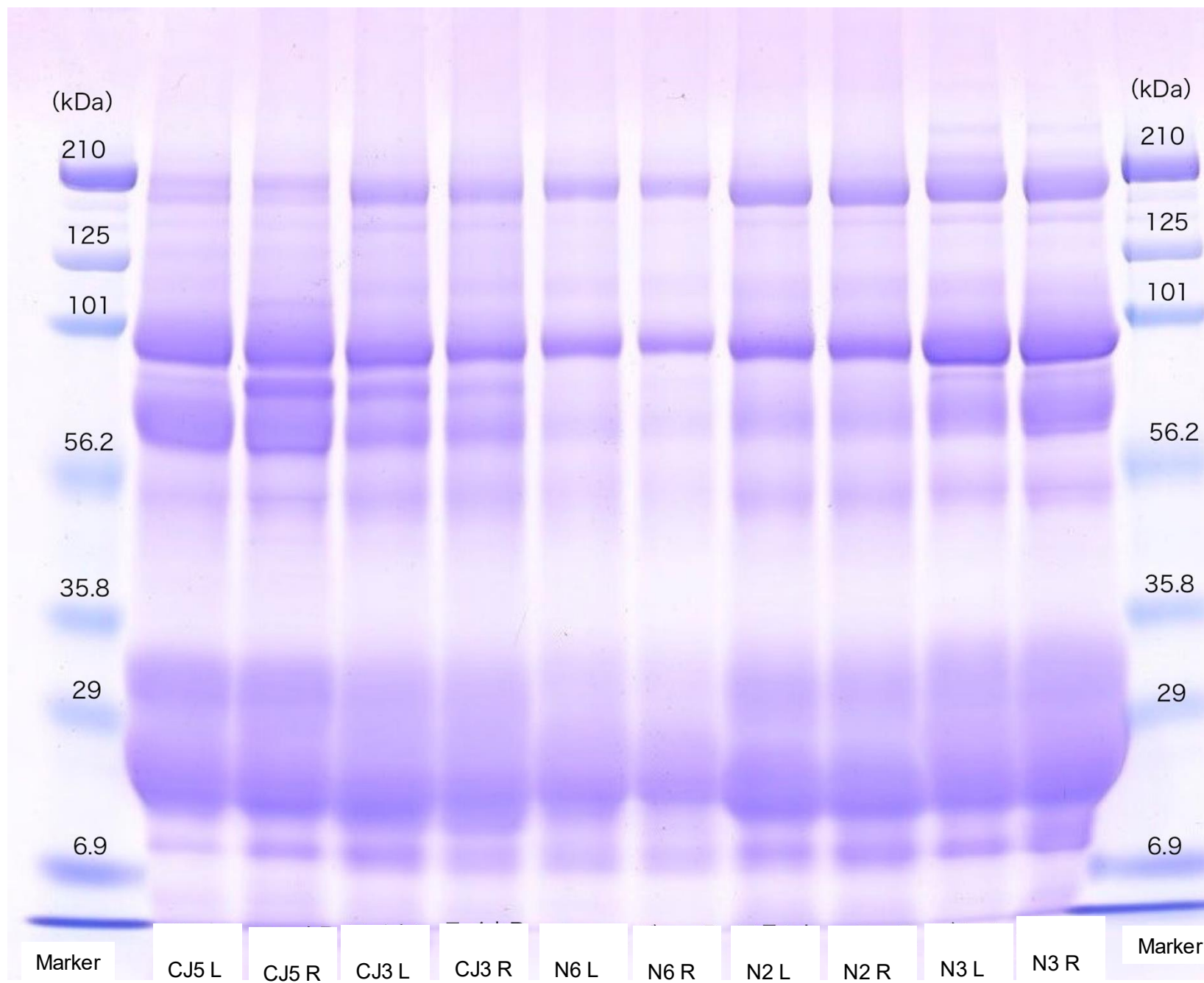

Supplement: Supplementary file 1 [file animals-16-00912-s001.zip › Supplimentary Figs.pdf]
